# Scallop-bacteria symbiosis from the deep sea reveals strong genomic coupling in the absence of cellular integration

**DOI:** 10.1093/ismejo/wrae048

**Published:** 2024-03-26

**Authors:** Yi-Tao Lin, Jack Chi-Ho Ip, Xing He, Zhao-Ming Gao, Maeva Perez, Ting Xu, Jin Sun, Pei-Yuan Qian, Jian-Wen Qiu

**Affiliations:** Department of Biology, Hong Kong Baptist University, Hong Kong SAR, 999077, China; Science Unit, Lingnan University, Hong Kong SAR, 999077, China; Institute of Evolution & Marine Biodiversity, Ocean University of China, Qingdao 266003, China; Deep-sea Science Division, Institute of Deep-sea Science and Engineering, Chinese Academy of Sciences, Sanya 572000, China; Department of Biology, Hong Kong Baptist University, Hong Kong SAR, 999077, China; Southern Marine Science and Engineering Guangdong Laboratory (Guangzhou), Guangzhou 511458, China; Department of Ocean Science, The Hong Kong University of Science and Technology, Hong Kong SAR, 999077, China; Institute of Evolution & Marine Biodiversity, Ocean University of China, Qingdao 266003, China; Southern Marine Science and Engineering Guangdong Laboratory (Guangzhou), Guangzhou 511458, China; Department of Ocean Science, The Hong Kong University of Science and Technology, Hong Kong SAR, 999077, China; Department of Biology, Hong Kong Baptist University, Hong Kong SAR, 999077, China

**Keywords:** chemosynthesis, cold seep, ectosymbiosis, glass scallop, thiotrophy

## Abstract

Previous studies have revealed tight metabolic complementarity between bivalves and their endosymbiotic chemosynthetic bacteria, but little is known about their interactions with ectosymbionts. Our analysis of the ectosymbiosis between a deep-sea scallop (*Catillopecten margaritatus*) and a gammaproteobacterium showed that bivalves could be highly interdependent with their ectosymbionts as well. Our microscopic observation revealed abundant sulfur-oxidizing bacteria (SOB) on the surfaces of the gill epithelial cells. Microbial 16S rRNA gene amplicon sequencing of the gill tissues showed the dominance of the SOB. An analysis of the SOB genome showed that it is substantially smaller than its free-living relatives and has lost cellular components required for free-living. Genomic and transcriptomic analyses showed that this ectosymbiont relies on rhodanese-like proteins and SOX multienzyme complex for energy generation, mainly on the Calvin–Benson–Bassham (CBB) cycle and peripherally on a phosphoenolpyruvate carboxylase for carbon assimilation. Besides, the symbiont encodes an incomplete tricarboxylic acid (TCA) cycle. Observation of the scallop’s digestive gland and its nitrogen metabolism pathways indicates it does not fully rely on the ectosymbiont for nutrition. Analysis of the host’s gene expression provided evidence that it could offer intermediates for the ectosymbiont to complete its TCA cycle and some amino acid synthesis pathways using exosomes, and its phagosomes, endosomes, and lysosomes might be involved in harvesting nutrients from the symbionts. Overall, our study prompts us to rethink the intimacy between the hosts and ectosymbionts in Bivalvia and the evolution of chemosymbiosis in general.

## Introduction

Chemosymbioses between lithoautotrophic bacteria and animals have been widely recognized as a driving force for the ecological adaptation and evolution of invertebrates ranging from sponges, sea anemones, flatworms, nematodes, arthropods, annelids, and molluscs [1-3]. Still, the factors that enable the initiation, maintenance, and development of chemosymbioses remain poorly understood [1].

Chemosymbiosis is most phylogenetically widespread in the molluscan class Bivalvia, with nine families spanning from the early branching protobranchs to the recent venerids known to host chemosynthetic bacteria [4-6]. The chemosymbionts of bivalves are diverse, with some being methane-oxidizing bacteria (MOB) and others sulfur-oxidizing bacteria (SOB) or both MOB and SOB [7]. They also exhibit different levels of intimacy with their hosts from loosely attached to the gill surfaces (e.g. thyasirids) to enclose within epithelial gill cells (e.g. vesicomyids) [3]. Bivalves adopt different modes of symbiont transmission—from environmental transmission to strictly maternal transmission through the eggs [8] and consequently, the symbionts display varying degrees of bottleneck-driven genome reduction [5, 7, 9, 10].

The prevalence and diversity of chemosymbioses in Bivalvia make them prominent models to investigate some of the key questions within the field of symbiosis research: (i) whether endosymbiosis gradually evolves from ectosymbiosis [11, 12] and (ii) whether vertical symbiont transmission evolves from horizontal symbiont transmission [9, 11]. Answering these questions will be facilitated by uncovering the full extent of the chemosymbiotic diversity in bivalves and resolving their evolutionary histories. Despite a growing body of research, we do not know whether symbiosis has evolved multiple times within the group or from a single common ancestor [13]. To this end, it is imperative to screen for chemosynthesis in the 113 currently recognized families of Bivalvia, especially in the majority (104 families) where no such symbiotic relationship has yet been reported [14].

Here, we report chemosymbiosis in the order Pectinida, a large group of Bivalvia commonly known as scallops and are widely present from shallow water to the deep sea, including chemosynthetic habitats [15-19]. The lack of morphological specializations in the gill of a vent-dwelling scallop has been suggested to indicate an absence of chemosymbiosis [16]. Yet, the discoveries of simple homorhabdic gills bearing chemosymbionts in thysarids [20] and a heterotrophic alphaproteobacterial endosymbiont in a shallow-water scallop [21] supported further investigation of chemosymbiosis in vent and seep scallops. *Catillopecten margaritatus*—a deep-sea glass scallop that inhabits the Haima seep in the South China Sea— is commonly found on the empty shells of the vesicomyid *Archivesica marissinica* and around the tubeworm *Sclerolinum annulatum* aggregation [22-24]. Our previous analyses of the stable isotopes of *C. margaritatus* [23] revealed δ^13^C and δ^34^S values typical of the SOB symbiont-bearing bivalves that are known to rely solely or mainly on SOB for nutrition [12, 23, 25]. In this study, we aimed to characterize the chemosymbionts of *C. margaritatus*. First, we used a combination of 16S rRNA gene amplicon sequencing and microscopic analyses to identify and locate the SOB. Then, we sequenced the genome of the SOB and compared it with those of other bivalve chemosymbionts. Lastly, we conducted *de novo* meta-transcriptomics to reconstruct the holobiont metabolism. Our results indicate that the gill of *C. margaritatus* hosts a single epibiont phylotype related to those associated with bathymodioline mussels but divergent to those of other co-occurring invertebrates. This symbiont is primarily reliant on thiosulfate for sulfide oxidation and lacks hydrogenotrophic capabilities. Besides, we found that despite the extracellular localization of the symbiont and the host's use of external food sources, its genome is relatively small compared with its free-living relatives and the level of host-symbiont metabolic complementarity was high. Together, these results suggest an obligate association between the host and the bacteria. Given that previous omics studies of symbiosis in Bivalvia have focused on endosymbionts with expected tight host-symbiont metabolic integration [5, 8, 10], our results are significant because they not only reveal a new evolutionary path from asymbiosis to symbiosis in scallops, but also open a gate for comparative studies of bivalve ectosymbionts that are widespread in Thyasiridae and small Bathymodiolinae [20, 26], and considered as the early stages of bivalve-chemosynthetic bacteria symbioses [4].

## Materials and methods

### Sample collection

Three *C. margaritatus* individuals were collected from the Haima seep (16°54.04’N, 110°28.47′E) during a dive by the remotely operated vehicle *Pioneer* on board research vessel (R/V) *Xiangyanghong 01* at 1433–1441 m water depth in September 2022. The samples were dissected onboard the R/V to separate the gill and gonad tissues, stored at −80°C for DNA and RNA sequencing, fixed by 4% paraformaldehyde (PFA) for hematoxylin–eosin (HE) staining and fluorescence in situ hybridization (FISH), and 2.5% glutaraldehyde for transmission electron microscopy (TEM), respectively.

### HE staining, FISH, and TEM observation

The HE staining and FISH were conducted to visualize the morphology and symbiont distribution in gill and gonad tissues from two specimens. The PFA-fixed gill and gonad samples were dehydrated with ethanol and soaked in xylene, then embedded in Paraplast (Sigma, USA). Paraffin block was cut into 6 μm sections using an RM 2126 microtome (Leica, Germany). Paraplast was removed using xylene and rehydrated and the sections were stained with hematoxylin (Abcam, UK) and observed under a differential interference microscope (Olympus BX51, Japan). For FISH, the rehydrated sections were treated with 0.1% tween 20 in phosphate-buffered saline (PBST) to increase permeability, and hybridized in formamide hybridization buffer (0.9 M NaCl, 0.02 M Tris–HCl, 0.01% sodium dodecyl sulfate (SDS), 35% deionized formamide, 0.5 μM of probe BangT-642 [27] labeled by Cy5 dye targeting thiotrophic Gammaproteobacteria and 0.5 μM of negative control probe IMedM-138 [27] labeled by Cy3 dye targeting methanotrophic Gammaprobacteria) for 1 h at 46°C. The sections were rinsed with washing buffer (0.1 M NaCl, 0.02 M Tris–HCl, 5 mM ethylenediaminetetraacetic acid (EDTA), 0.01% SDS) for 15 min at 48°C, and stained using 4',6-diamidino-2-phenylindole (DAPI, Sigma, USA) for 3 min at room temperature. Then the sections were embedded in Antifade Mounting Medium (Beyotime, China) under a cover slip. The sections were observed under an LSM 710 NLO laser scanning confocal microscope (ZEISS, Germany).

For TEM observation, the gill sample preserved in glutaraldehyde was washed in PBS, transferred to 1% osmic acid for further fixation at 4°C for 2 h, rinsed in PBS, then dehydrated in a gradient of ethanol solutions and embedded in epoxy resin (EPON 812). After polymerizing at 37°C, 45°C, and 65°C for 24 h, respectively, the resin blocks were cut into 70 nm ultrathin sections with an EM UC7 ultramicrotome (Leica, Germany), and then stained by uranyl acetate then by lead nitrate and observed with a JEM-1200EX transmission electron microscope (JEOL, Japan).

### DNA extraction, 16S rRNA gene amplicon analysis, and phylogenetic reconstruction

Bacterial 16S rRNA gene amplicon sequencing for the gill samples were conducted to determine the bacterial composition. Genomic DNA was extracted from three individuals using the cetyltrimethylammonium bromide (CTAB) method [28]. The V3–V4 region of the bacterial 16S rRNA gene was amplified with the primers 341F and 806R targeting bacteria [29, 30], and the libraries were generated using a TruSeq DNA PCR-Free Sample Preparation Kit (Illumina, USA). Sequencing was conducted on a NovaSeq6000 platform (Illumina, USA) under the PE250 mode in Novogene (Tianjin, China), generating 84 443, 92 176, and 93 095 raw reads, respectively (Table S1).

The 16S rRNA gene amplicon analysis was performed using the QIIME2 v2023.9 pipeline [31] to generate an amplicon sequence variants (ASV) table. The adapters were removed using the cutadapt trim-paired command. Paired-end sequence reads were merged using the vsearch’s merge_pairs function and filtered using the quality-filter q-score command. The Deblur workflow was employed for sequence quality control, utilizing a 16S rRNA gene reference as a positive filter. Reads were classified by taxon using the Greengene2 2022.10 (https://forum.qiime2.org/t/introducing-greengenes2-2022-10/25291) and visualized using the taxa barplot command to generate a barplot of bacterial abundances.

Phylogenetic analysis for the 16S rRNA gene sequences of the dominant and unique SOBs of the scallop and the symbiotic bacteria of related hosts (Table S2-S3) was performed using PhyloSuite v1.2.2 [32] to determine its phylogenic position. MAFFT v7.520 [33] was applied under the “auto” option to align gene fragments. Gblocks v0.91b [34] was applied to remove ambiguously aligned fragments in batches. The Bayesian Inference (BI) analysis was performed using MrBayes v3.2.6 [35] implemented in PhyloSuite for 10 million generations, with the initial 25% of the sampled data discarded as burn-in, and the best-fit substitution model GTR + I + Γ + F determined by ModelFinder implemented in PhyloSuite based on the Bayesian information criterion (BIC). The maximum likelihood (ML) analysis was performed using IQ-TREE v2.1.2 [36] implemented in PhyloSuite under the best-fit substitution model TIM3 + I + Γ4 + F selected by ModelFinder and ran for 1 000 ultrafast bootstraps.

### DNA library construction and metagenomic sequencing

Total DNA samples of the gill (G3) containing the host and symbiont DNA, and the gonad tissues (SG1 and SG2, Table S1) were used for the construction of whole-genome shotgun libraries with an insert size of 350 bp, using NEBNext Ultra DNA Library Prep Kit for Illumina (NEB, USA). The libraries were sequenced on a NovaSeq 6000 sequencer (Illumina, USA) in Novogene (Tianjin, China). A Nanopore library of G3 was constructed using a Ligation Sequencing Kit 1D (PM) following the manufacturer's protocol. The library was sequenced on an Oxford Nanopore PromethION platform in Novogene (Tianjin, China), with 1.0 μg of the prepared library loaded onto a FLO-PRO002 flow cell (ID: FLO-PRO002).

### Genomic assembly, mapping, annotation, and phylogeny

The adaptors and low-quality Illumina reads were removed using Trimmomatic v0.39 [37] (settings: LEADING:15 TRAILING:15 SLIDINGWINDOW:4:20 MINLEN:40). The Nanopore reads were base-called using Guppy v6.3.9 under the high-accuracy mode, and the reads were corrected and trimmed using Canu v2.1.1 [38] under the default settings, and then assembled using Canu with the genome size and maxInputCoverage set to 1.4 Mb (estimated based on the assembly of Illumina reads) and 1200, respectively. A single, circular bacterial genome was obtained, and two sequential rounds of error correction against the trimmed reads were applied using Minimap2 v2.27 [39] and Racon v1.4.13 [40] under the default settings. Then, the polished genome and the Illumina reads were aligned using BWA v0.7.17 [41], parsed using Samtools v1.12 [42], and base-call polishing using Pilon v1.23 [43] with a mindepth of 1. The genome quality was estimated using CheckM2 v1.0.1 [44], and the genome was annotated using Prokka v1.13.4 [45]. Then Pfam, COG, GO, and KEGG annotations were conducted using eggNOG-MAPPER v5.0 [46] against the eggNOG HMMs database. Besides, KEGG Mapper [47] was used to reconstruct the metabolic pathways. The average nucleotide identity (ANI) values among the selected genomes were calculated using JspeciesWS [48] to determine their phylogenetic distances. To further demonstrate the absence of symbiont in the gonad, metagenomic sequencing reads of two gonad tissue samples were also assembled and binned like above, and mapped to the symbiont genome using Bowtie2 v2.3.3.1 [49].

Phylogenomic analyses were performed as in a pipeline [50], including orthologous groups (OGs) identification using Orthofinder v2.2.7 [51], sequence alignment using MAFFT v7.520 [33], trimming using Gblocks v0.91b [34], and removal of paralogues using Phylopyprunner (https://gitlab.com/fethalen/phylopypruner). Protein sequences from the *C. margaritatus* symbiont genome, 24 available symbiotic or free-living SOB genomes, and two outgroups were used (Table S3). Two matrices (50% and 80% orthologue occupancy) were prepared. Single-copy OGs were sorted using GenesortR [52], and 600 OGs were selected to build the ML phylogenetic tree using IQ-Tree 2 v2.1.2 [36] under the MFP option for model selection and then run for 1 000 ultrafast bootstraps [53]. To compare the genomic structures of the *C. margaritatus* symbiont and its close relatives, Mauve v2.4.0 [54] was applied with a match seed weight of 15 and a minimum LCB score of 30 000.

### RNA extraction, library construction, and metatranscriptomic sequencing

Metatranscriptomic sequencing was applied to quantify the symbiont gene expression levels and recover the host transcriptome. The total RNA of three gill samples (G1-G3) was extracted using the Trizol reagent (TAKARA, Japan). The metatranscriptomic sequencing was performed in Novogene (Tianjin, China). Briefly, ribosomal RNA was removed using NEBNext Ultra RNA Library Prep Kit for Illumina (NEB, USA), and RNA molecules were fragmented into 250–300 bp and reverse-transcribed into cDNA. The libraries were sequenced on a NovaSeq 6000 sequencer (Illumina, USA) to produce approximately 63.2 million 150-bp paired-end reads per sample (Table S1).

### *De novo* metatranscriptomic assembly and analyses

The adaptors and low-quality reads of Illumina sequencing were removed using Trimmomatic v0.39 [37] (settings: LEADING:15 TRAILING:15 SLIDINGWINDOW:4:20 MINLEN:40). The clean reads were used for de novo meta-assembly using Trinity v2.8.5 [55] under the default settings. *De novo* assembly generated 972 872 transcripts. The protein-coding genes (PCGs) were predicted using TransDecoder v5.5.0 [55] under the default settings. The highly redundant, bacterial, and potential contamination transcripts were removed. Briefly, Cd-Hit-Est v4.7 [56] was applied to remove redundant contigs using 95% as the sequence similarity cutoff. The PCGs were BLASTx searched against the NR database with an E-value threshold of 1e-10 using DIAMOND v0.9.24 [57], and the bacterial and other potential contaminant hits were removed to generate the host transcripts. After the filtering, 41 024 unigenes were retained. Gene annotation was conducted as in section 2.5, and a total of 25 473 (62.1%, the final host transcriptome) were annotated against at least one public database. A BUSCO v5.4.2 [58] analysis with the Metazoan_odb10 database showed that the transcriptome contained 87.5% complete (including 1.5% duplicated) and 5.6% fragmented metazoan BUSCOs. To quantify the gene expression, the clean reads were mapped to the host transcriptome and symbiont genes under the default settings, respectively, and expressed as transcripts per million (TPM) using Salmon v0.14.1 [59]. Besides, the Pearson correlation was used to evaluate the consistency in gene expression among the three transcriptome samples, which showed a high consistency between G2 and G3 (*r* = 0.94), but very low consistency between G1 and G2 (*r* = 0.02) and G1 and G3 (*r* = 0.10). The RNA data of sample G1 was not used for further analysis due to its low quality (Table S1). The final gene expression levels were determined using the average TPM values of G2 and G3. The host and symbiont genes with TPM values larger than 100 and 300, respectively, were defined as highly expressed genes (HEGs) [6]. The KEGG enrichments of these HEGs were conducted using TBtools-II v2.008 under the default settings [60].

## Results and discussion

### Symbionts are thiotrophic and located outside the scallop’s gill cells

**Figure 1:**
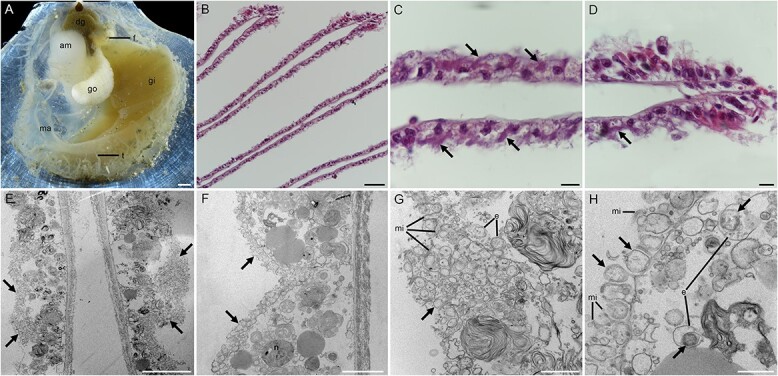
Anatomy features of *Catillopecten margaritatus* and photomicrographs showing the extracellular localization of the SOB. A: Anatomy features of *C. margaritatus* showing the gill tissue, left valve. B-D: Hematoxylin–eosin (HE) stain of gill sections showing a gross view of the gill filaments. E-H: Transmission electron microscopy (TEM) of gill filament showing the extracellular distribution of the SOBs. The bacteria are in the extracellular spaces filled by microvilli, indicated by arrows. Scale bar: A: 1 mm; B: 50 μm; C-D: 10 μm; E: 10 μm; F: 5 μm; G: 2 μm; H: 1 μm. Am, adductor muscle; b: Bacteria; dg, digestive gland; e: Endocytosis; f, foot; go, gonad; gi, gill; ma, mantle; mi: Microvillus; t, tentacle.

**Figure 2:**
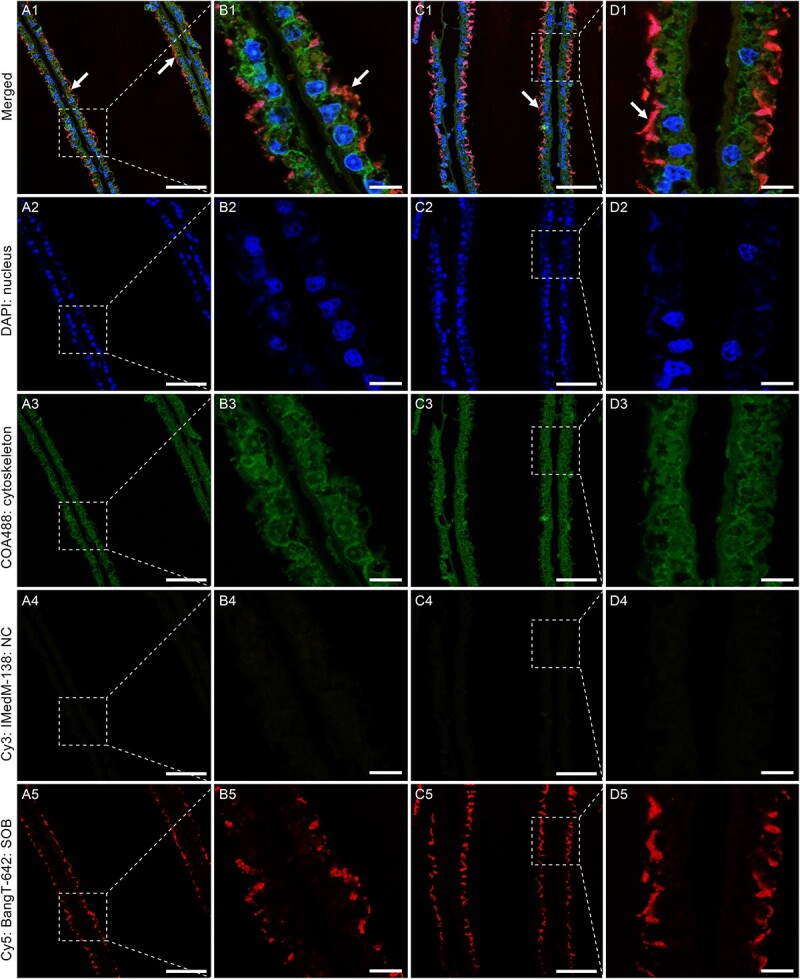
Fluorescence in situ hybridization (FISH) showing the extracellular localization of the SOB. The DAPI channels show the locations of the nuclei and the COA488 channels represent the gill cytoskeleton. The Cy3 channels show the negative control (NC) using the IMedM-138 probe targeting methanotrophic Gammaproteobacteria. The Cy5 channels indicate the bacteria based on the BangT-642 probe targeting thiotrophic Gammaproteobacteria (SOB). The bacteria are indicated by arrows, and the weak bacterial signals inside the gill cells might be the ectosymbionts endocytosed by the host, as indicated by the TEM micrographs (Fig. 1G and H). Scale bar: A & C: 50 μm; B & D: 10 μm.

Our HE staining analysis of the gill sections revealed abundant basophilic particles (deep purple colour indicating DNA) on the surface and between the microvilli of the gill epithelial cells (Fig. 1B-D). Our TEM analysis confirmed aggregations of bacteria on the surface and between the microvilli of the gill epithelial cells (Fig. 1E-H). The observed spherical bacteria were not methanotrophs as they did not contain intracellular concentric stacks [61]. Besides, we found endosomes containing bacterial cells in some sections (Fig. 1G and H), indicating that the host may harvest the symbionts by endocytosis. Our FISH analysis, using a fluorescent probe specific for thioautotrophic Gammaproteobacteria and a negative control targeting methanotrophic Gammaproteobacteria [27], confirmed that they belong to sulfur-oxidizing Gammaproteobacteria and further supported their extracellular distribution on the host’s gill epithelial cells (Fig. 2). Overall, these observations indicate that the symbionts of *C. margaritatus* are extracellular sulfur-oxidizing Gammaproteobacteria associated with its gill epibacteriocytes. We also conducted FISH analysis of the gonad but did not detect any SOB signal (Fig. S1), which suggests that the symbionts may not be vertically transmitted via germ cells. This observation is consistent with the dominance of horizontal symbiont transmission mode in chemosynthetic ectosymbionts [62]. Among bathymodioline mussels, both extracellular and intracellular symbioses were identified, with *Gigantidas* species hosting intracellular MOB [10], *Bathymodiolus* species hosting intracellular SOB and/or MOB [63], and small bathymodiolines like *Adipicola*, *Idas*, and *Nypamodiolus* harboring ectosymbiotic SOB [26, 64]. Previous studies hypothesized that these small bathymodiolines were the intermediate forms between their asymbiotic shallow-water ancestors and the bigger deep-sea vent- and seep-species adopting intracellular symbiosis [12, 65]. Therefore, our discovery of ectosymbiosis in *C. margaritatus* provides a model to test the hypothesis of symbiont acquisition during the shallow-water to deep-sea transition in bivalves.

### Scallop gill harbors one dominant thiotrophic symbiont strain

**Figure 3:**
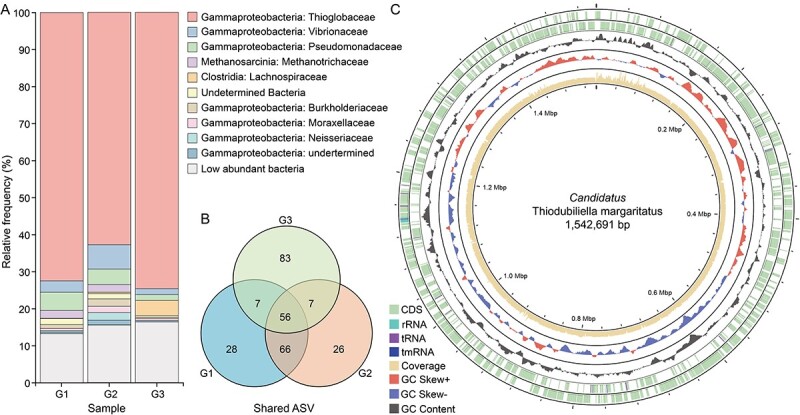
Gill bacterial community composition of *Catillopecten margaritatus* and genomic overview of the ectosymbiont *Candidatus* Thiodubiliella margaritatus. A: 16S rRNA gene amplicon sequencing results showing the relative frequency of the SOB symbiont and other bacteria in three individuals at the family level. The most abundant amplicon sequence variant (ASV) is a sulfur-oxidizing bacteria from the family Thioglobaceae (Table S4). B: Venne diagram showing the shared ASVs among the three samples. C: Circus plot of the ectosymbiont genome with its features. From the outer to inner circle: CDS, contigs, GC content, GC skew, genomic size.

A microbial 16S rRNA gene amplicon analysis revealed the dominance of a thiotrophic gammaproteobacterium belonging to the genus *Thiodubiliella* in the three samples, accounting for 72.5%, 62.8%, and 74.6% of the total reads, respectively (Fig. 3A and B, Table S4). Of the less abundant bacteria, we identified Desulfobacterota, Campylobacterota, and methane-oxidizing Proteobacteria with the ASV number of 18, 5, and 1, respectively (Table S4). Some of these chemosynthetic bacteria were also identified from the animal and sediment samples collected from the Haima seep [10, 66, 67], and most of them were present in only one of the three samples with low abundance (<1%) (Table S4), indicating they are environmental contaminants. Our phylogenetic analyses showed that the dominant thiotrophic gammaproteobacterium was nested in a clade containing mainly gill symbiotic SOB of bathymodiolines (Fig. S2).

### Glass scallop’s ectosymbiont is absent in the gonad and phylogenetically close to bathymodioline symbionts

*De novo* assembly using both Nanopore and Illumina reads recovered a complete and circular gammaproteobacterial genome in a single contig measuring 1.54 Mb (Fig. 3C; Table S5). The genome is substantially smaller than those of the free-living SOB *Thiomicrospira crunogena* [68] and the environmentally acquired intracellular bathymodioline SOB [10], but only slightly smaller than that of the *B. septemdierum* and *Conchocele bisecta* symbionts which were considered in an intermediate state between extra- and intracellular symbiosis [69, 70]. CheckM2 analysis showed that it had 99.99% completeness and 0% contamination, indicating its high quality compared to other published SOB genomes (Table S3). Mapping the Illumina reads to the symbiont genome showed that it represented 43.3% of the reads from the gill sample, indicating that the symbiont is abundant on the gill surface, consistent with microscopic observation (Figs 1 and 2). Gene prediction showed that the symbiont genome contained 1 577 protein-coding genes (PCGs), 3 rRNAs, 36 tRNAs, and 1 tmRNA. The PCGs were well annotated (1 527 PCGs, 96.8%). The COG annotation indicated that the functional composition of the scallop symbiont genome was similar to that of bathymodioline SOB (Table S6).

*De novo* assembly and binning using Illumina reads of gonad tissues (Table S1) did not generate any bacterial genome. The mapping of Illumina reads to the ectosymbiont genome produced above showed that only a few ectosymbiont reads could be identified in the gonad samples (218 and 684 in 34.81 and 38.34 million reads, respectively). The proportion of ectosymbiont reads in the gonad was extremely small (about 0.001% on average) compared to the gill (43.3%), indicating these gonad-associated reads were contaminants and the ectosymbiont is not vertically transmitted via germ cells.

**Figure 4:**
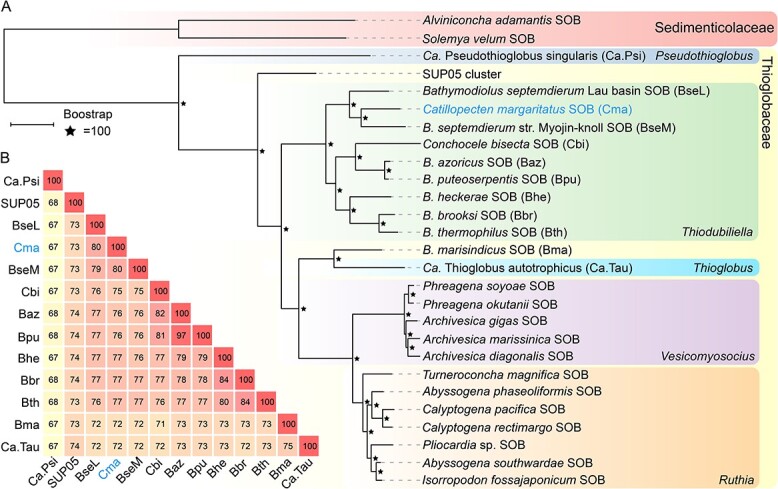
Phylogenetic relationships and genetic distances between the scallop ectosymbiont and other symbiotic and free-living sulfur-oxidizing bacteria (SOB). A: Maximum-likelihood tree constructed using 600 single-copy orthologs, with the endosymbionts of *Solemya velum* and *Alvinconcha adamantis* as the outgroups (Table S3). The scale bar (0.1) indicates the mean number of amino acid substitutions per site. B: The ANI values between the ectosymbiont of *Catillopecten margaritatus* and its relatives. The symbiont names are abbreviated based on their host’s names indicated in A.

Consistent with the 16S rRNA gene amplicon results, phylogenetic analyses of 27 SOB genomes (Table S3) showed that this scallop symbiont belongs to the genus *Thiodubiliella* of the family Thioglobaceae (Fig. 4A), being sister to the SOB symbionts of *Bathymodiolus* and most closely related to those of *B. septemdierum* (Fig. 4A). Nevertheless, the symbiont genome of *C. margaritatus* and the two closest symbiont genomes of *B. septemdierum* had ANI values of ~80% only (Fig. 4B) – much lower than the recommended threshold of 95% for conspecific bacteria [71], indicating that these symbionts belong to different species. Therefore, we proposed *Candidatus* Thiodubiliella margaritatus as the name for the SOB symbiont of *C. margaritatus*. Besides bathymodiolines, symbiotic *Thiodubiliella* is also known to associate extracellularly with the vent thyasirid clam *C. bisecta* [69]. These symbionts are closely related to those of *B. azoricus* and *B. puteoserpentis* (Fig. 4), underscoring the genus’s ability to form a broad range of associations with bivalves. *Catillopecten margaritatus* usually lives on the empty shells of vesicomid *A. marissinica* or the sediment around aggregations of the tubeworm *S. annulatum* [24]. Although they co-occur, the SOB symbionts of these three species showed huge phylogenetic divergences [5, 22, 24], which may reflect their distinct evolutionary histories and subtle physiological differences allowing them to exploit sulfur resources in the heterogeneous habitats.

### Scallop and bathymodioline symbiont genomes are structurally divergent yet contain conserved central metabolism blocks

**Figure 5:**
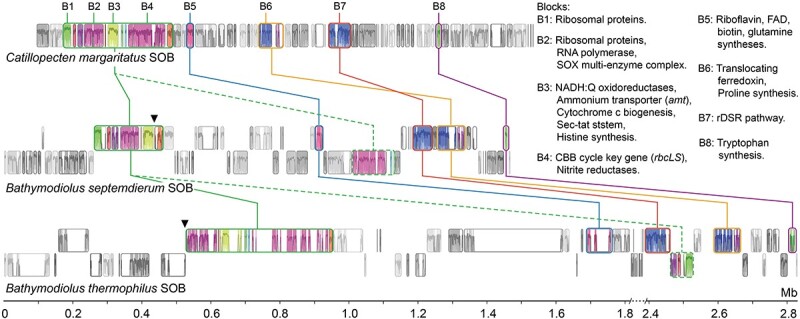
Conserved gene blocks among the symbionts of *Catillopecten margaritatus* and its two closely related bathymodioline symbiont genomes**.** The diagram is based on whole genome alignment, with the largest conserved blocks as an anchor. The crucial genes and pathways located in the conserved blocks were indicated at the upper right. The inverse or transposed blocks are labeled using dashed lines, and their original locations are indicated by triangles.

Whole genome alignment revealed different genomic structures among Ca. *T. margaritatus* and two available complete bathymodioline symbiont genomes, with multiple insertions, translocations, and inversions among them (Fig. 5; Fig. S3; Table S7). This contrasts with vesicomyid symbiont genomes which are highly conserved in genomic structure, except for a single block of 20 genes/pseudogenes missing in one clade and present in the other [5, 9, 72]. Nevertheless, the Ca. *T. margaritatus* genome contained eight conserved blocks (B1–8), including sulfur oxidation pathways – the SOX multienzyme complex and reverse dissimilatory sulfite reduction (rDSR) pathway (B2 & B7). The high conservation of these genomic blocks among the SOB symbiont genomes of the three bivalve species indicates selection may have favored the preservation of their organization due to their conserved roles in energy generation. Besides, large and small subunits of RuBisCO form I (*rbcLS*)—the key genes of the Calvin-Benson-Bassham (CBB) cycle—and several nitrite reductases were also conserved in block B4 between Ca. *T. margaritatus* and *B. thermophilus* SOB, whereas this block was inversely translocated in *B. septemdierum*. Several other genes and pathways of critical functions, including genetic expression (B1–2), ammonium transporter (B3), and some nutrient biosynthesis pathways (B3, B5–6, & B8), were also identified in the conserved blocks (Fig. 5; Table S7).

### Chemosynthetic capabilities of the scallop ectosymbiont

**Figure 6:**
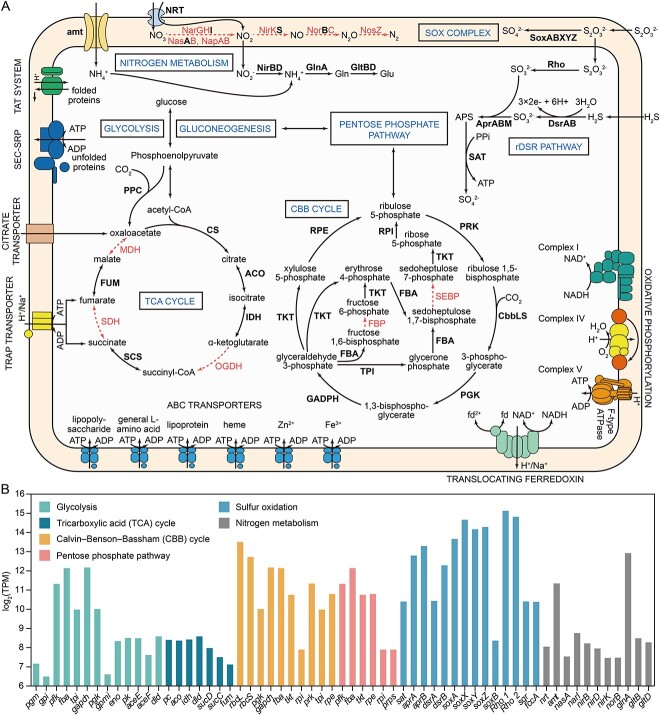
Predicted metabolic map and gene expression levels of the ectosymbiont associated with *Catillopecten margaritatus*. A: Putative metabolic map of Ca. *T. margaritatus*. The pathways and enzymes are indicated by solid arrows and bold font, and the missing pathways and enzymes are indicated by dashed arrows. B: Transcriptional levels of the central metabolic pathways, expressed as log_2_-transferred average transcripts per million (log_2_(TPM)) values (Table S9).

Chemosynthesis is crucial for many deep-sea vent and seep holobionts. Although the genome of Ca. *T. margaritatus* is substantially smaller than the bathymodioline SOBs, they all encode the core genes and pathways for carbon fixation and energy production (Fig. 6A, Table S8–9).

#### Sulfur metabolism is primarily reliant on thiosulfate oxidation

**Figure 7:**
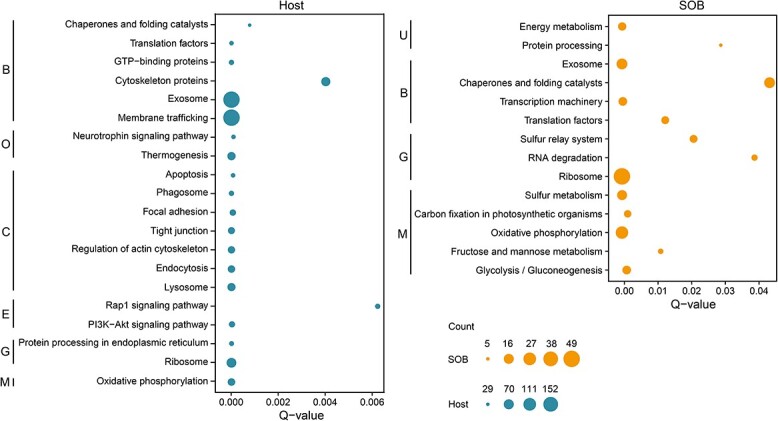
Functional enrichment of highly expressed genes (HEGs) in the gill of host and ectosymbiont. Only pathways with a Q-value <0.05 were considered significant. Only the top 20 abundant pathways of the host were presented (Table S11). The count refers to the number of genes in each category. B: Brite hierarchies; C: Cellular processes; E: Environmental information processing; G: Genetic information processing; M: Metabolism; O: Organismal systems; U: Unclassified.

All transcriptomic analyses were based on two replicates (G2 and G3, Table S1). The KEGG enrichment of the highly expressed genes showed that the scallop’s ectosymbiont was actively engaged in sulfur metabolism and carbon fixation (Fig. 7; Tables S10–S11). Significantly, sulfur oxidation was the most highly expressed metabolic process (Fig. 6B), with two rhodanese-like proteins suggested to catalyze thiosulfate to sulfite [7, 73] ranked the top second and third in transcription in the scallop’s ectosymbiont (Fig. 6; Table S10). The sulfite produced by rhodanese-like proteins can be further oxidized by the adenylylsulfate reductase (AprAB) (Fig. 6A). Besides, the genes of L-cysteine S-thiosulfotransferase (*soxAX*) and sulfur-oxidizing protein (*soxYZ*) were also highly expressed, ranked after rhodanese-like proteins, and the SOX multienzyme complex was more active than the rDSR pathway (Fig. 6B; Table S10). Both the SOX multienzyme complex and rDSR pathway are widely used for sulfur oxidation by the SOBs of deep-sea bathymodioline mussels [74-76], vesicomyid clams [5, 77], and siboglinid tubeworms [78, 79]. The relative transcriptional levels of these two systems differ among these symbionts, which might be related to their different habitats and material sources. Those SOBs associated with animals capable of obtaining hydrogen sulfide from the sediment tend to show high transcriptional of the rDSR pathway, such as those of the clams *A. marissinica* [5] and *Solemya velum* [77], and the tubeworms *Riftia pachyptila* [78], *Paraescarpia echinospica* [79], and *S. annulatum* [24]. By contrast, the SOX multienzyme complex, utilizing thiosulfate from ambient water, is more transcriptionally active than rDSR pathway in bathymodioline symbionts [7]. Therefore, the ectosymbiont of *C. margaritatus* living on the seafloor or the shells of *A. marisinica* has the potential to use thiosulfate from seawater instead of sulfide from sediment, indicating its physiological adaptation to the availability of sulfur in the environment.

#### Carbon is assimilated via the CBB cycle supplied by a phosphoenolpyruvate carboxylase

The CBB cycle is used by most autotrophs for carbon fixation. In Ca. *T. margaritatus*, the CBB cycle was transcriptionally active, as shown by the high expressional levels of *rbcLS*, glyceraldehyde 3-phosphate dehydrogenase (*gadph*), and fructose bisphosphate aldolase (*fba*) (Figs 6B and 7; Table S10). Similar to the endosymbiotic SOB of other bivalves [5, 6, 8], *fpb* is absent in the genome of Ca. *T. margaritatus*. Nevertheless, the CBB cycle is functional and active in the symbiont for fixing carbon dioxide and provision of intermediates and biomass for the holobiont. In addition, the reductive tricarboxylic acid (rTCA) cycle—another carbon assimilation pathway in prokaryotes—is incomplete in Ca. *T. margaritatus*, with most of the genes missing. However, phosphoenolpyruvate carboxylase (*ppc*)—the key gene of the rTCA cycle—is identified in the genome of Ca. *T. margaritatus* (Table S9), which encodes a carboxylase that catalyzes the carboxylation of phosphoenolpyruvate to oxaloacetate and fixes carbon dioxide simultaneously [80]. This gene was lowly expressed with an average TPM value of 56, compared to the high level of the key genes *rbcL* (TPM = 11 642) and *rbcS* (TPM = 6 765) in the CBB cycle. Therefore, *ppc* may play a subsidiary role in inorganic carbon assimilation for the ectosymbiont.

#### Holobiont may require filter-feeding to meet its nitrogen requirement

The thiotrophic symbionts of vesicomyids, bathymodiolines, and siboglinids encode complete dissimilatory nitrate reduction pathways to provide electron accepters and ammonia [6, 24, 74-76, 79]. Our previous study found that the δ^15^N value of *C. margaritatus* (9.9‰) was substantially higher than those of clams, mussels, and tubeworms reliant on SOB for nutrients only [23]. Because our observation of the scallop showed that it had a full digestive gland (Fig. 1A), it is likely mixotrophic, relying partially on filter-feeding to meet its nutritional requirements. Filter-feeding has also been suggested to be responsible for a part of the nutrition of the deep-sea bathymodioline mussels [81] and anemones [82] hosting SOB symbionts. We found that Ca. *T. margaritatus* genome encodes an incomplete dissimilatory nitrate reduction pathway for nitrite reduction producing ammonium, and an ammonium transporter to intake ammonium from ambient water (Fig. 6A). Most of the genes involved in nitrogen metabolism, including the key genes nitrate/nitrite transporter (*nrt*) and nitrite reductase (NADH) large and small subunits (*nirBD*), were lowly expressed (Fig. 6B). By contrast, ammonium transporter (*amt*) and glutamine synthetase (*glnA*) were transcriptionally active, with TPM values of 2 605 and 7 770, respectively (Fig. 6B), indicating that ectosymbiont is more dependent on ammonium from the water column for glutamine biosynthesis than on nitrite reduction. In addition, two nitroreductases with TPM values of 392 and 189 were identified, indicating that this scallop SOB may have the ability to degrade toxic nitro-containing compounds like trinitrotoluene [83] from the environment to obtain additional nitrogen. Therefore, *C. margaritatus* may be mixotrophic, relying on both chemosynthesis and filter-feeding to obtain nitrogen materials. Further investigation is desired to pinpoint the nitrogen sources and nitrogen assimilation pathways in the scallop holobiont.

#### Ectosymbiont lacks the hydrogenotrophic capability

The fluids from vents typically have higher hydrogen concentrations than those from seeps [84, 85], therefore it has been hypothesized that this energy source is a driver for the differences in chemosynthetic bacteria between the two habitat types, with certain bacteria from hydrothermal vents possessing hydrogenase for energy production. This may explain why the SOB symbionts of some chemosynthetic invertebrates (i.e. mussels *B. septemdierum*, *B*. *puteoserpentis*, and *B*. *thermophilus*) inhabiting vents encode hydrogenases [74, 86, 87], whereas seep-dwelling clam *Thyasira* sp. and tubeworm *S. annulatum* lack these genes [6, 24]. In line with this hypothesis, we found that the genome of Ca. *T. margaritatus* lacks hydrogenases. Nevertheless, a [NiFe] hydrogenase was found in the SOB symbionts of the seep tubeworm *P. echinospica* [79]. Therefore, more chemosymbionts should be screened for the presence/absence of hydrogenases to better understand their roles in the evolution of chemosynthesis.

### Ectosymbiosis is probably obligate and the host and symbiont are metabolically interdependent

Obligate symbiotic bacteria are typically small and lack genes and pathways essential for free living, unlike facultative symbionts [88, 89]. For instance, the obligate endosymbionts of the vesicomids are only ~1 Mb in genome size and have lost many genes involved in cellular envelope, motility, and heavy metal resistance [5, 9]. In the Ca. *T. margaritatus* genome, key genes for the flagellum, pilus, and chemotaxis systems are also missing (Fig. 6A; Tables S8–S9), indicating that the ectosymbiont lacks motility and environmental sensing. Although the ectosymbiotic Ca. *T. margaritatus* is not transmitted vertically via germ cells, the missing of these genes does not allow this symbiont to maintain an extended free-living period in the ambient water, as indicated for the ectosymbiont symbionts of thyasirids where these components essential for free-living are also absent [6, 69]. Therefore, we suggest that Ca. *T. margaritatus* is likely an obligate symbiont transmitted horizontally. This is possible as obligate symbionts have been shown capable of surviving outside the host for a short period [90], and horizontal transmission of obligated symbionts have been reported in several groups of marine invertebrates [91, 92]. For instance, whole-genome analyses of *S. velum* revealed signatures of frequent horizontal transmission of its obligate symbionts [93]. In vent tubeworms *R. pachyptila*, *Oasisia alvinae*, and *Tevnia jerichonana*, obligate endosymbionts colonize the developing tube of the settled larvae and horizontally enter the host through the skin [94]. Besides, the horizontally transmitted thiotrophic symbiont of *B. azoricus* is suggested to be an obligate symbiont [7]. Therefore, it is possible that the ectosymbionts of *C. margaritatus* disperse and colonize the scallop larvae in the water column within a short period of their release from the host. That the scallops usually live in aggregations could provide such opportunities for close contact [22].

Previous studies of chemosynthesis have revealed tight metabolic interdependence between the hosts and endosymbionts [5, 8, 79]. Here, we showed that the metabolic complementarity between a deep-sea scallop and its ectosymbiotic SOB could be more intimate than previously thought. In the scallop’s ectosymbiont, the TCA cycle is incomplete without malate dehydrogenase (*mdh*), succinate dehydrogenase/fumarate reductase (*sdh*), and 2-oxoglutarate dehydrogenase (*ogdh*). Previous studies have found the absence of these genes in the intracellular symbiont of the mussel *B. azoricus* [7] and the ectosymbiont of the clam *Thyasira* sp. [6], and thus they could not synthesize oxaloacetate, fumarate, and succinate. In *B. azoricus*, these intermediates could be supplied from the host cytosol via intaking by C4-dicarboxylate transporter (TRAP transporter) and citrate transporter [7]. We found these transporters in the Ca. *T. margaritatus* genome and they were highly expressed (480, 202, 3 300, and 568 TPM for *DctQ*, *DctM*, *DctP* subunits of the TRAP transporter, and citrate transporter respectively, Table S9), which indicates that these missing intermediates of the symbiont’s TCA cycle must be supplied exogenously, except oxaloacetate may be compensated by *ppc*. Although the extracellular location of Ca. *T. margaritatus* prevents it from accessing the intermediates in the host’s cytosol directly, our enrichment of HEGs in *C. margaritatus* gill tissues showed that the genes involved in exosome and membrane trafficking were highly expressed, being the top two enriched pathways (Fig. 7). Exosomes are extracellular vesicles carrying bioactive molecules like DNA, RNA, and functional proteins, which play significant roles in intercellular communication and immune response [95]. In human-associated bacteria, exosomes can take up many different TCA cycle intermediates including succinate and fumarate from the host [96]. Thus, we propose that exosomes and membrane trafficking genes are involved in the transport of succinate and fumarate from the host’s gill cytosol to the microvillus space, which are then taken up by the ectosymbiont to complete its TCA cycle via transporters. Nevertheless, further evidence is needed to support this hypothesis.

Deep-sea molluscs hosting endosymbionts usually lose the capability to synthesize some amino acids and cofactors [6, 10, 79]. We examined the scallop’s transcriptome and the ectosymbiont genome to investigate their nutrient biosynthetic capabilities (Table S12). Our analysis of the host’s transcriptome showed that the biosynthetic pathways of 12 amino acids (asparagine, aspartate, chorismate, histidine, leucine, lysine, ornithine, phenylalanine, proline, and tryptophan) and eight cofactors (FAD, folate, lipoic acid, pantothenate, protoheme, riboflavin, pyridoxine phosphate, and ubiquinone) were incomplete (Fig. S4; Table S12). These results were only based on the gill transcriptomic data with moderate completeness (87.5% of complete metazoan BUSCOs), and it is desirable to analyze the host’s genome to reveal its metabolic potentials. Nevertheless, the ectosymbiont genome encodes the genes for biosynthesis of all these nutrients (except for ubiquinone) (Fig. 7). The symbionts could be captured by the host via endocytosis or phagocytosis, as indicated by our microscopic observation (Fig. 1F and G), and enrichment and high expression of many genes related to the phagosome, endocytosis, and lysosome in the host gill (Fig. 7). By contrast, the Ca. *T. margaritatus* genome lacks some genes essential for the biosynthesis of cysteine, methionine, and threonine (Fig. S4; Table S12), indicating that the symbiont might obtain them from the host. This might be carried out by the host's exosomes, as indicated by the expression of the related genes (Fig. 7). However, exactly how these nutrients are transported from the host to symbionts requires further study.

## Conclusion

We provided microscopic and molecular evidence for scallop-associated thiotrophic bacterial ectosymbiont. This ectosymbiont is phylogenomically related to the thiotrophic symbionts of bathymodiolines, but their genomic structures are substantially different. Because its TCA cycle, several amino acid biosynthetic pathways, secretion system, and motility system are incomplete, it is likely an obligate symbiont, dependent on the host to provide some of the missing nutrients. The ectosymbiont’s rhodanese-like proteins and SOX multienzyme complex were highly expressed in energy generation, consistent with sulfur oxidization as its main source of energy. Its CBB cycle was also highly expressed, indicating this is the primary pathway for inorganic carbon assimilation. Nevertheless, we also found evidence that its incomplete rTCA cycle plays a subsidiary role in inorganic carbon assimilation. The host might obtain energy and nutrients from the symbionts by harvesting them via phagosomes, endosomes, and lysosomes, whereas providing specific amino acids and some metabolic intermediates to the symbiont. The discovery of this obligate ectosymbiosis with a tight host-bacteria metabolic complementarity not only enables comparative genomic studies to test various hypotheses on the evolution history of symbiosis in Bivalvia but also prompts more multi-omics research on the roles of ectosymbionts in host nutrition.

## Supplementary Material

Supplementary_figures_wrae048

Supplementary_tables_wrae048

## Data Availability

The data sets presented in this study can be found in online repositories. All amplicon, Illumina, Nanopore, RNA sequencing data, symbiont genome, and its annotation were deposited in the National Centre for Biotechnology Information (NCBI) under the BioProject PRJNA1029732. We have released all the data submitted to NCBI and Figshare. The functional annotations and expressional levels were deposited in Figshare under DOI: 10.6084/m9.figshare.24406429.
