# Supplementary material for: Scallop-bacteria symbiosis from the deep sea reveals strong genomic coupling in the absence of cellular integration
Source: ISME J. 2024 Mar 26;18(1):wrae048. doi: 10.1093/ismejo/wrae048 (PMC10999363; doi:10.1093/ismejo/wrae048)
Supplement: Supplementary_figures_wrae048 [file supplementary_figures_wrae048.docx]

**Supplementary figures**

**Scallop-bacteria symbiosis from the deep sea reveals strong genomic coupling in the absence of cellular integration**

Yi-Tao Lin^1^, Jack Chi-Ho Ip^2^, Xing He^3^, Zhao-Ming Gao^4^, Maeva Perez^1^, Ting Xu^5,6^, Jin Sun^3^, Pei-Yuan Qian^5,6^, Jian-Wen Qiu^1,*^

^1^Department of Biology, Hong Kong Baptist University, Hong Kong SAR, China

^2^Science Unit, Lingnan University, Hong Kong SAR, China

^3^Institute of Evolution & Marine Biodiversity, Ocean University of China, Qingdao 266003, China

^4^Institute of Deep-sea Science and Engineering, Chinese Academy of Sciences, Sanya 572000, China

^5^Southern Marine Science and Engineering Guangdong Laboratory (Guangzhou), Guangzhou 511458, China

^6^Department of Ocean Science, The Hong Kong University of Science and Technology, Hong Kong SAR, China

^*^Correspondence: Jian-Wen Qiu, E-mail: [qiujw@hkbu.edu.hk](mailto:qiujw@hkbu.edu.hk); Address: Department of Biology, Hong Kong Baptist University, RRS820, 224 Waterloo Road, Kowloon Tong, Hong Kong SAR, China


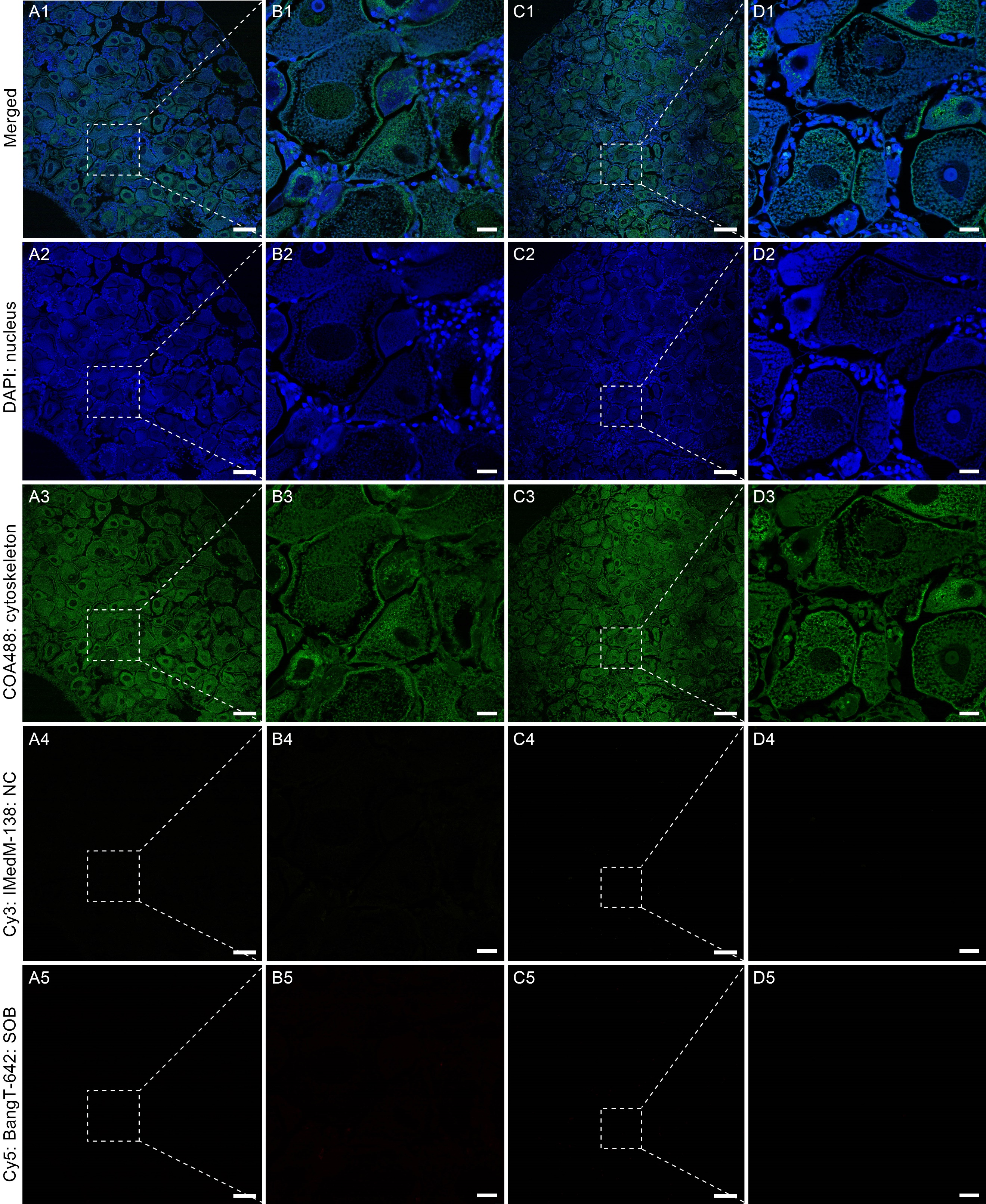


**Fig. S1 Fluorescence in situ hybridization (FISH) showing the absence of the SOB in the gonad tissue.** The blue signals show the locations of the nucleus and the green signals represent the gill cytoskeleton labeled by COA488. The yellow signals show the negative control (NC) using the IMedM-138 probe labeled by Cy3 targeting methanotrophic Gammaproteobacteria, while the red signals indicate the bacteria (white arrows) based on the BangT-642 probe labeled by Cy5 targeting thiotrophic Gammaproteobacteria (SOB). Scale bar: A & C: 100 μm; B & D: 20 μm.


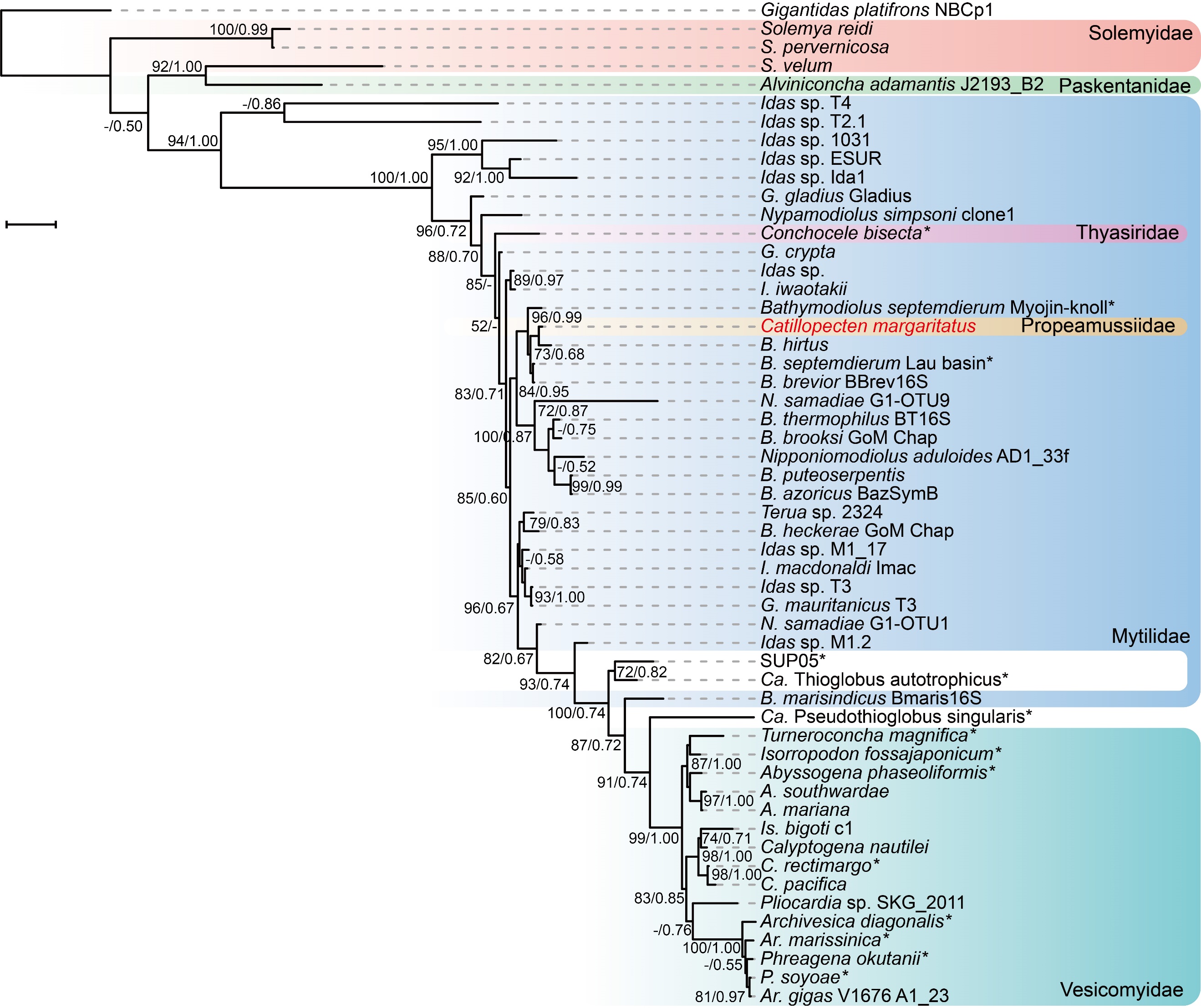


**Fig. S2 Phylogenetic relationships among the gill-associated bacteria of *Catillopecten margaritatus* and other SOB relatives inferred from the *16S rRNA* gene fragments.** The topology is based on Bayesian inference (BI) analysis using a 1,537 bp matrix, with the methane-oxidizing symbiont of *Gigantidas platifrons* as the outgroup (Table S2-3). The scale bar (0.02) indicates the mean number of amino acid substitutions per site. Bootstraps (BP) values of the maximum-likelihood (ML) analysis and posterior probabilities (PP) of the BI analysis are shown near the nodes. The hosts from different families are indicated by different colors. The sequence obtained from this study is indicated in red.


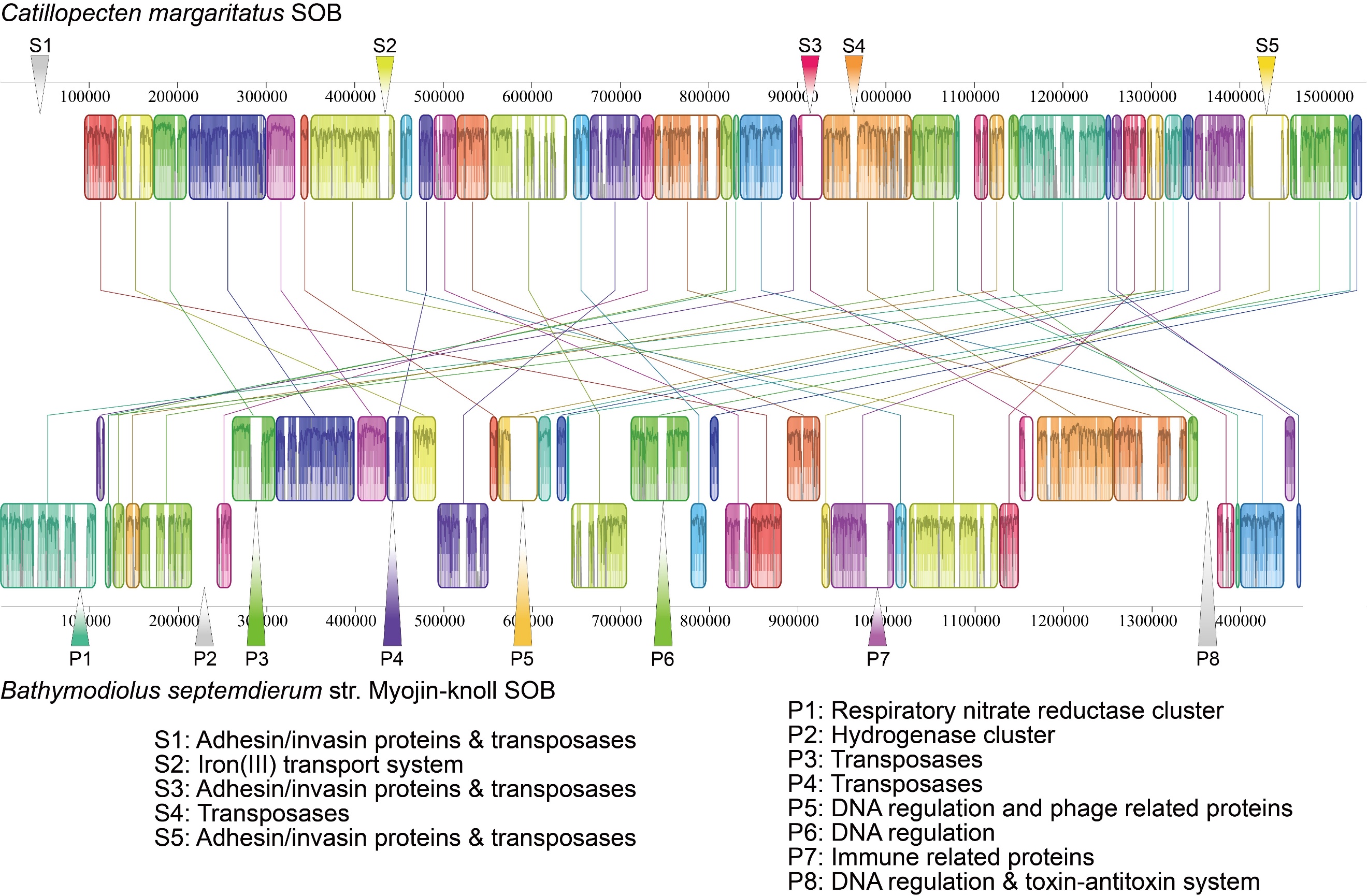


**Fig. S3 Whole genome alignment between the symbionts of *Catillopecten margaritatus* and *Bathymodiolus septemdierum*.** Different colours show different alignment blocks and the misalignment areas were indicated using triangles (sites S1-S5 for *C. margaritatus*, positions P1-P8 for *B. septemdierum*) (Table S8).


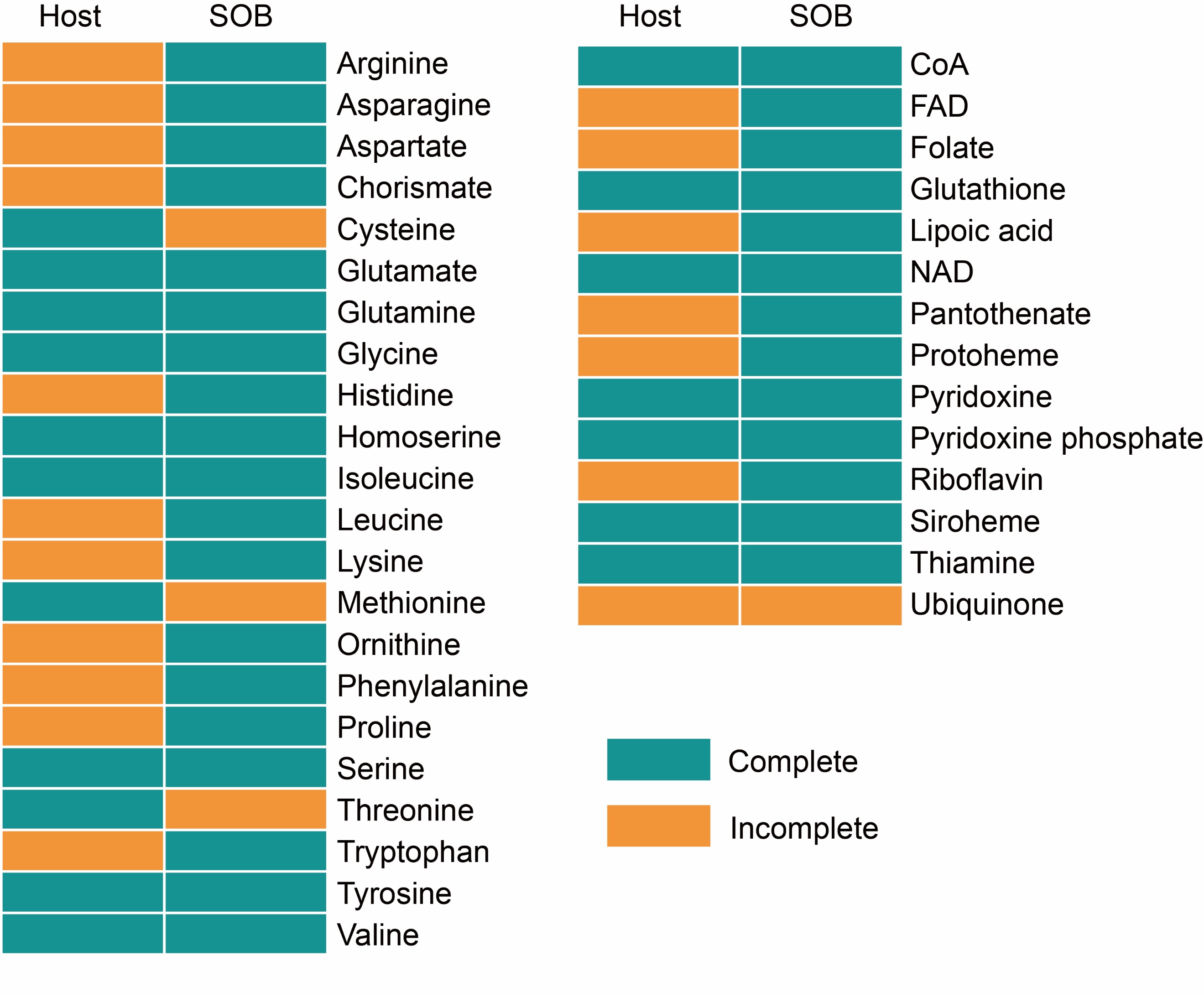


**Fig. S4 Pathways related to** **the biosynthesis of amino acids and cofactors found in the *Catillopecten margaritatus* gill transcripts and its ectosymbiont (Table S12).**
